# Development of a Personalized Mobile Mental Health Intervention for Workplace Cyberbullying Among Health Practitioners: Protocol for a Mixed Methods Study

**DOI:** 10.2196/23112

**Published:** 2020-11-20

**Authors:** Yun Jin Kim, Linchao Qian, Muhammad Shahzad Aslam

**Affiliations:** 1 School of Traditional Chinese Medicine Xiamen University Malaysia Sepang Malaysia

**Keywords:** workplace cyberbullying, mental health, personalized mobile mental health intervention, health informatics, Malaysia

## Abstract

**Background:**

Workplace cyberbullying harms the psychological and social functioning of professionals working in an organization and may decrease the productivity and efficiency of daily life tasks. A recent study on trainee doctors across 8 different United Kingdom National Health Service trusts found health issues and job dissatisfaction in people who have experienced workplace cyberbullying. This disabling effect is even more noticeable in low-socioeconomic communities within low-income countries. In Malaysia, there is a need to create a personalized mobile mental health intervention program for health care professionals. These programs should be directed to prevent and decrease psychosocial issues and enhance coordination among health care professionals to solve health issues in the community.

**Objective:**

Our main objective is to study the pre-effects and posteffects of the Personalized Mobile Mental Health Intervention (PMMH-I) for workplace cyberbullying in public and private hospitals in Malaysia.

**Methods:**

A hospital-based multimethod multi-analytic evidential approach is proposed, involving social and psychological health informatics. The project has been subdivided into 3 stages, starting with Phase 1, a prevalence study, followed by exploratory studies. Phase 2 consists of a quasi-experimental design, whereas the development of a prototype and their testing will be proposed in Phase 3. Each stage includes the use of quantitative and qualitative methods (mixed-method program), using SPSS (version 26.0; IBM Corp) and Stata (version 16.1; StataCorp) as tools for quantitative research, and NVivo (version 1.0; QSR International) and Atlas.ti (version 9.0.16; ATLAS.ti Scientific Software Development GmbH) for qualitative research.

**Results:**

The results of this study will determine the pre- and posteffectiveness of an integrated PMMH-I for health care professionals. The prototype system platform will be developed and implemented in a public and private hospital. Results from Phase 1 will be published in 2021, followed by the implementation of Phase 2 in subsequent years.

**Conclusions:**

This study will provide evidence and guidance regarding the implementation of a personalized mobile mental health intervention for health care professionals into routine public and private hospitals to enhance communication and resolve conflicts.

**International Registered Report Identifier (IRRID):**

PRR1-10.2196/23112

## Introduction

### Background

Suicidal behavior, depression, loneliness, anxiety, and somatic symptoms are among the most common effects of cyberbullying experienced by students [1] and adolescents [2]. There is a huge psychological impact (ie, depression) on people who experience workplace bullying from coworkers, such as nurses [3-6], health care providers, and employers [7], especially among women. The prevalence of cyberbullying among first-year trainee doctors who reported experiencing it at least 1 time was 46.2% [8]. Another study found a high prevalence rate (28%) of cyberbullying among a diverse workforce sample in the United States [9].

Malaysia has a high mobile penetration rate due to thriving e-commerce [10]. The problematic use of smartphones can lead to addiction; increased use of mobile phones in India has led to an increase in technology addiction [11,12]. This addiction leads to depression, anxiety, stress, and self-esteem issues [13]. A school-based study identified a strong correlation between pathological internet use and cyberbullying in Greece [14]. However, there is a lack of evidence that pathological mobile use could lead to an increase in cyberbullying in the workplace. Most published studies, systematic reviews, and interventions are limited to cyberbullying at school, colleges, universities, or the young population.

### Research Questions

We aim to explore the following 4 research questions in this study: (1) What is the current workplace cyberbullying status among Traditional Chinese Medicine practitioners (TCM) in Malaysia? (2) What is the pre- and postefficacy of the Personalized Mobile Mental Health Intervention (PMMH-I) on the prevalence of measurable factors (ie, depression, anxiety, and stress) in workforce cyberbullying on TCM practitioners? (3) What are the moderating and mediating factors of workplace cyberbullying on TCM practitioners? (4) What is the technological feasibility of implementing the integrated PMMH-I as a component of routine hospital-based systems?

### Main Intention and Objective

This study's main intention is to give awareness and authority to the community of health care professionals against cyberbullying by developing a Personalized Mobile Mental Health Intervention program (PMMH-I) in partnership with all health care providers. This partnership will seek to form alliances with public and private institutions to ensure the success and continuation of the program. The PMMH-I program will aim to reduce the psychometric properties of cyberbullying through awareness and adaptation of technology.

The main objective of this protocol is to develop and implement the PMMH-I program to decrease the psychometric properties of cyberbullying in the hospital-based setting in Malaysia. We hypothesize that the creation of PMMH-I programs will come about by executing a PMMH-I strategy that permits the fusion of global and local knowledge. PMMH-I promotes health care community development, thereby increasing social integration between different health care providers such as doctors, nurses, traditional and complementary medical practitioners, pharmacists, and other health care providers.

### Theoretical Approach

**Figure 1:**
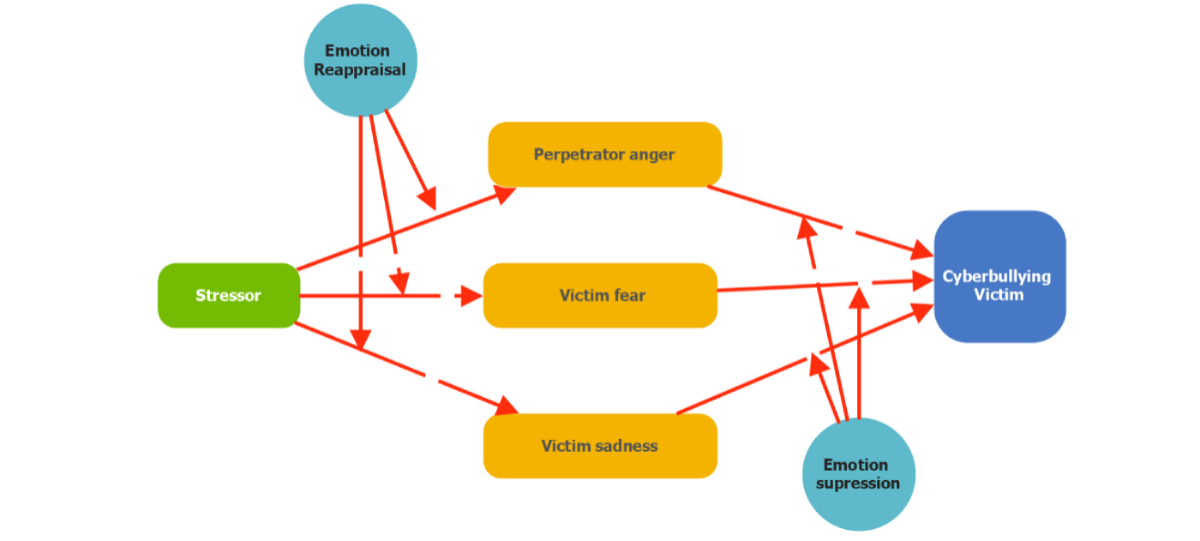
A proposed theoretical model of the emotion reaction model.

We will adopt the emotion reaction model (ERM) [15] as our theoretical framework, which allows researchers to explore the relationships among concepts of stressor, emotions, emotion regulators, and people who experience cyberbullying in the hospital setting in Malaysia. The ERM elucidates how emotions may produce an impact on people who experience cyberbullying in the current technological era. The adaptation of ERM depicted in Figure 1 posits the relationship between stressor, emotions, and people who experience cyberbullying, which is mediated by emotion reappraisal and emotion suppression.

## Methods

### Ethics Approval

According to the local and national ethical instructions for research (the National Committee for Clinical Research) guidelines, this study did not require ethics approval.

### Data Sharing Statement

No data are available. All data relevant to the study are included in the manuscript.

### Study Plan

**Figure 2:**
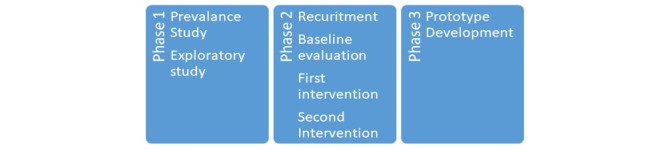
Research flow diagram.

**Figure 3:**
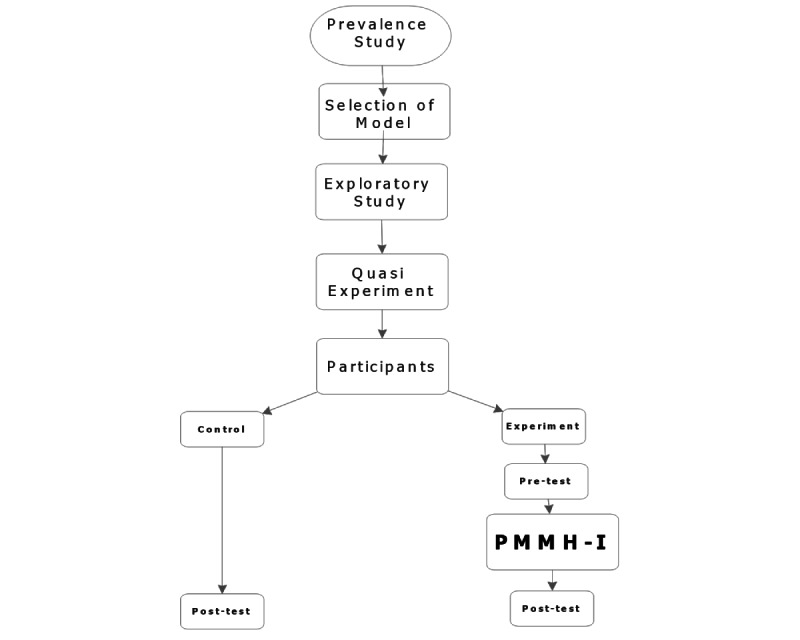
Flow diagram of the experimental design; PMMH-I: Personalized Mobile Mental Health Intervention.

**Figure 4:**
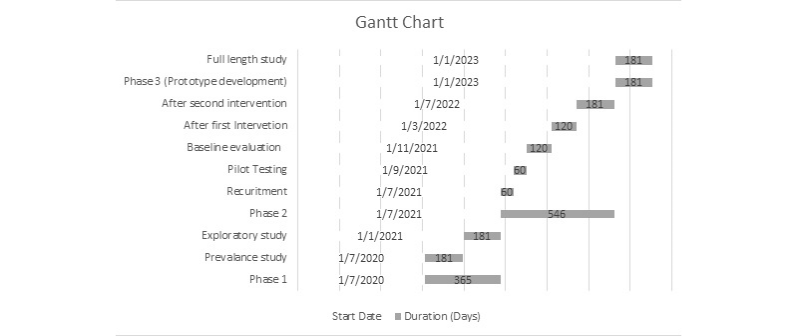
Gantt chart of study-related activities.

A hospital-based multimethod multi-analytic evidential approach is proposed, involving social and psychological health informatics. The project has been subdivided into 3 stages, starting with Phase 1, a prevalence study, followed by exploratory studies. Phase 2 consists of a quasi-experimental design, whereas the development of a prototype and their testing will be proposed in Phase 3. Flow diagrams for the research design are presented in Figures 2 and 3. A breakdown of our anticipated timeline for each phase of the study appears in Figure 4.

Each stage includes the use of quantitative and qualitative methods (mixed-method program), using SPSS (version 26.0; IBM Corp) and Stata (version 16.1; StataCorp) as tools for quantitative research, and NVivo (version 1.0; QSR International) and Atlas.ti (version 9.0.16; ATLAS.ti Scientific Software Development GmbH) for qualitative research.

### Phase 1: Multimethod Multi-Analytic Evidential Approach

#### Objectives

Phase 1 will consist of the identification and analysis of the problem. The duration of this phase will be 6 months and will include knowledge development about workplace cyberbullying among TCM practitioners.

#### Prevalence Study

The specific objectives of this study include (1) evaluating the overall prevalence rate of workplace cyberbullying and its related psychological impacts (depression, anxiety, and stress) on TCM practitioner; (2) understanding the measures associated with various forms of workplace cyberbullying on age, gender, and frequency; and (3) understanding the measure associated with workplace cyberbullying on perpetrators responsible for workplace cyberbullying. The current research will be a survey-based online cross-sectional prevalence study. TCM practitioners are considered a hard-to-reach population; thus, we will conduct a nonprobability snowball sample of 1023 adults (3% precision, 95% confidence level) according to the number of TCM practitioners, which was approximately 15,000 in 2011 [16]. The Hamilton Depression Scale (HAM-D) [17], the Hamilton Anxiety Scale (HAM-A) [18], and the clinical anger scale [19] will be used to assess the psychological impact on TCM practitioners. The Cyberbullying Questionnaire (CBQ) and a short version of a cyberbullying behavior questionnaire (CBQ-S), adapted from Jönsson et al [20], will be used to identify the prevalence among TCM practitioners; these selected questionnaires were already validated in Sweden and the US adult population [20]. The prevalence will also investigate the association with moderating variables (age and gender). Perpetrators of cyberbullying will also be assessed in the specific identification of the prevalence of workplace cyberbullying using the CBQ and CBQ-S questionnaires. The sampling method to understand perpetrator effects in the prevalence will similarly be addressed. The survey will also include a socioeconomic assessment, including education, income, home characteristics, and commodities. The survey will be conducted online due to the current pandemic situation caused by COVID-19, following the CHERRIES guidelines given by Gunther Eysenbach [21].

#### Exploratory Study

The purpose of the exploratory study is to understand the moderating and mediating factors of workplace cyberbullying by using the ERM on TCM practitioners [15]. The specific questions of this study include the following: (1) What are the relationships between emotions, workplace stressors, cyberbullying perpetration, cyberbullying victimization, and control appraisal, reappraisal, and suppression? (2) What effects do demographic factors have on emotions, workplace stressors, cyberbullying perpetration, cyberbullying victimization, and control appraisal, reappraisal, and suppression? The survey will be conducted using a convenience method, with a sample size of 384 (5% precision, 95% confidence level). A structural equation model will be developed, and each construct will be discussed. A snowball sampling method will be used to approach the target population. There will be a lucky draw at the end of the exploratory study on the participants; the 10 winners of the draw will receive an electronic power bank.

This procedure was selected due to a hard-to-reach study population and a study undertaken during the COVID-19 pandemic.

### Phase 2: Program Development

#### Objectives

**Figure 5:**
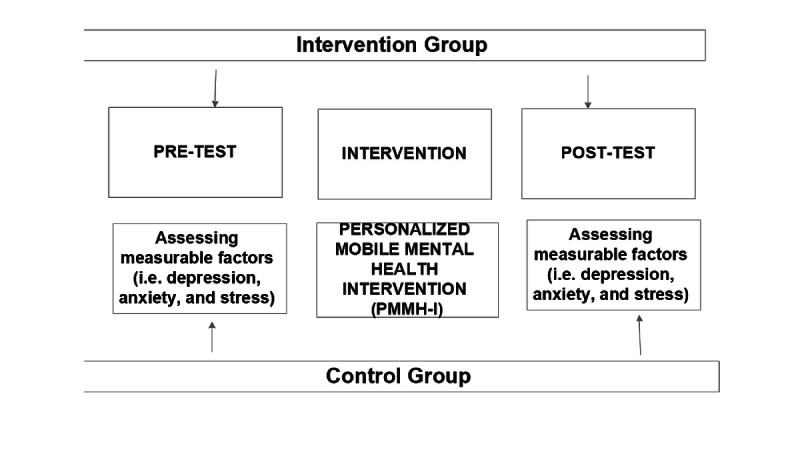
Overview of the nonequivalent control-group pretest–post-test study.

The aim of Stage 2 is to develop the anticyberbullying intervention program. Past approaches have been ineffective because of a lack of consistency within the community. To achieve this objective, a quasi-experiment with a nonequivalent pretest–post-test control group design will be conducted for a duration of 1-1.5 years (Figure 5).

#### Quasi-experiment with Nonequivalent Pretest–Post-test Control Group Design

The study design for this stage is quasi-experimental, in which the groups are nonrandomized into intervention and control groups and selected purposively. This study will be conducted in public hospitals and private hospitals located in Malaysia. All hospitals are eligible to participate. The selection of hospitals will be randomized for the implementation of the interventions. The randomization of hospitals will be performed by online statistical computing web programming to generate the randomization schedule [22]. The current methodology will help reduce selection bias in the study.

This intervention study will be conducted on TCM practitioners specifically. A baseline assessment will be done, in the intervention and control hospital, to measure the burden of bullying and its correlates using the CBQ and CBQ-S. This stage will begin after the investigation of prevalence and exploratory research. First, baseline information will be collected within 3 months, and then the intervention will be monitored twice after a regular period of 6 months in order to monitor the consistency of the intervention program.

The intervention program will be designed and finalized after (1) a systematic review of systematic reviews of previously published literature on bullying and the effectiveness of antibullying intervention programs; (2) the conducting of focus group discussions with TCM practitioners, patients, and other health care providers (to explore the perceptions, beliefs, and suggestions regarding bullying prevention programs); and (3) the conducting of a consultation workshop with all the stakeholders. The bullying intervention program (BIP) will pretest among 50 participants, which will include health care providers, TCM practitioners, and patients. The multicomponent hospital-based intervention strategy will be administered at 3 levels: (1) patients, (2) TCM practitioners, and (3) other health care providers. All the data of participants in this study will be password-protected and safe using Microsoft OneDrive. The recruitment of participants will follow the same procedure described for the exploratory study and prevalence study.

### Phase 3: Electronic Anticyberbullying Monitoring System

#### Interface Design

An expert from each stakeholder group (TCM practitioners, other health care professionals, patients, and IT experts) will be involved in the interface design, information system design, and prototype testing. The system’s overall performance and satisfaction will be acquired through questionnaires (questionnaires are available upon request). The CBQ and CBQ-S will be used to determine the effectiveness of an intervention program using a prototype (post-test). The compilation of this research will be used to design the Personalized Mobile Mental Health Intervention (PMMH-I). The duration for stage 3 will be 6 months.

#### Literature Review on Interventions for Workplace Cyberbullying

There is limited literature available on interventions for workplace cyberbullying among TCM practitioners. Most of the available literature is limited to the identification of exploratory factors, measurement factors, and increasing trends of workplace cyberbullying. Funded by the Massey University Research Fund, D’Souza et al [23] discussed the issue of workplace cyberbullying in New Zealand; the study sample size was limited to 20 participants, but they predict an increase of workplace cyberbullying [23,24]. Vranjes et al [25] developed the inventory of cyberbullying acts at the workplace (ICA-W); the available results are preliminary items and build upon existing knowledge.

Moreover, the results of available literature are often generalized, noncontextual, and lack practical implications [25]. Another online self-report survey on workplace cyberbullying in New Zealand reports an increasing trend in workplace cyberbullying [26]; however, self-reported answers may be exaggerated and lead to an increase in survey bias. The studies of Farley [27], who had worked on the workplace cyberbullying measurement scale, are limited to methodological improvement in the measurement scale; these studies call for further exploratory studies. Other studies on the exploratory factors of workplace cyberbullying [27-29] lack coordination between theoretical and practical applications. The subjects of cyberbullying literature in Malaysia are limited mostly to adolescents and undergraduate students, and the focus is primarily on prevalence, the determination of exploratory factors, and descriptive studies [30-33]. The available studies are limited to examining the exploratory factors of workplace bullying [34].

Our studies will adopt the ERM in hospitals, and will adapt personalized mobile-based IT consumerization of the ERM in workplace cyberbullying among health care professionals as the intervention. A recent study also supports mobile phone– and internet-based interventions for treating depression [35]. Our study will be the first to use a personalized mobile mental health intervention for workplace cyberbullying for TCM practitioners.

#### Data Collection and Analysis

The recruitment of participants will follow the same procedure as was described for the exploratory study and prevalence study. The participant will be invited to join through a webinar to complete the questionnaire. Only the participants who completed Phase 2 will be asked to continue. The prototype will be given to them, and a brief training will be provided through a 30-minute webinar. Stage 3 participants will be limited to the Stage 2 completers in order to gauge the effectiveness of the Step 2 intervention program and whether the prototype is capable of reporting cyberbullying effectively. Thematic analysis will be done on the data yielded from the control and intervention groups. All the data of participants in this study will be password-protected and safe using Microsoft OneDrive.

## Results

The study was submitted for funding by an internal grant from Xiamen University Malaysia in May 2020. The prevalence study for Phase 1 began in July 2020, and it will be followed by exploratory research in January 2021. The duration for Phase 1 will be 12 months. For Phase 2, the recruitment of participants will begin in July 2021. The baseline study will be completed in 4 months. The intervention will be divided into two 4-month periods to measure the consistency of the intervention. The last phase will be the development and implementation of the prototype with pilot testing and post-testing, which will have a duration of 6 months (subject to the availability of funding).

## Discussion

The impetus for this study is a need for technologically based interventions to decrease work-related cyberbullying identified by health professionals and academics. Workplace cyberbullying leads to counterproductive work behaviors [36] and may lead to violence due to conflicts or work-related stress. This study will also provide insight into how job-related stress (role conflicts), team-related stress (interpersonal conflicts), and organization-related stress (organizational change) can lead to unintentional cyberbullying communication. This causes cyberbullying victimization between doctors, traditional alternative practitioners, and other health care providers. There is a lack of evidence on knowledge generation indicators, such as prevalence studies. To reduce conflict and enhance work productivity, there is a need for exploratory studies to understand the ERM and the adaptation of technological interventions such as personalized mobile mental health in hospital-based settings.

## Appendix

Multimedia Appendix 1 Peer Review Report.

## Abbreviations

CBQ: Cyberbullying Questionnaire
CBQ-S: short version of a cyberbullying behavior questionnaire
ERM: emotion reaction model
ICA-W: inventory of cyberbullying acts in the workplace
PMMH-I: Personalized Mobile Mental Health Intervention program
TCM: Traditional Chinese Medicine
