# Supplementary material for: Development of a Personalized Mobile Mental Health Intervention for Workplace Cyberbullying Among Health Practitioners: Protocol for a Mixed Methods Study
Source: JMIR Res Protoc. 2020 Nov 20;9(11):e23112. doi: 10.2196/23112 (PMC7718091; doi:10.2196/23112)
Supplement: Multimedia Appendix 1 [file resprot_v9i11e23112_app1.pdf]

XIAMEN UNIVERSITY MALAYSIA
厦門大學馬來西亞分校

Xiamen University Malaysia Research Fund (XMUMRF)
Evaluation Form

Year	2020	Cycle	6

Name of evaluator	
Title/designation	
Department/Faculty/University	
Home/Office address	
Mobile phone no	
Email	
Principal Investigator (PI)	Dr. Aslam Muhammad Shahza
Title of proposed research project	Development of Personalized Mobile Mental Health Intervention for Workplace Cyberbullying on Health practitioners

Evaluation

			Y/N	Comment
1	Research project team			
	PI	Appropriate qualifications to conduct and complete the proposed research	Y	PI has the foundation of previous work.
	Co-researchers (if applicable)	Appropriate qualifications	Y	Experts in Chinese medicine
2	Title of proposed research project	Appropriate for the proposed research activities, etc	Y	The title appropriately explains the research content.
		Not appropriate, too general, etc		
3	Research area/field	Appropriate or not	Y	Mental Health
4	Duration	Appropriate or not	Y	Suitable
5	Abstract	Well written, informative, etc	Y	Concise and accurate
		Poor, recommend to rewrite, etc		
6	Objective(s)	Clearly written, achievable, etc	Y	cyberbullying in public and private hospitals
		Ambiguous, etc		
		Too ambitious, etc		

7	Literature review/ Research background	Concise, acceptable with some relevant key references, etc	N	The relevant key references and research background are less
		Irrelevant, insufficient, etc		
8	Research methodology	Good, using existing methods, etc		
		Good, developing new methods, etc	Y	Be going to develop new methods
		Insufficient details, etc		
9	Flowchart	Lucid, informative, etc	N	No flowchart
10	Expected results or benefits	Reasonable, achievable, etc	Y	Reasonable
11	Benefits of the proposed research to XMUM, the economy, and the society	Reasonable, achievable, etc	Y	Solve mental issues.
12	Comment on the status of similar or related research in Malaysia and abroad; how is the proposed research different from them	Well written, informative, etc	N	Nill
13	Project schedule/Gantt chart	Reasonable, achievable, etc	Y	perfect
14	Project milestones	Lucid, achievable, etc	Y	Clearly and completely
15	Budget			
	Allowance for assistant	Reasonable, comply with Guidelines		Not mentioned
	Travel and subsistence	Reasonable, comply with Guidelines		Not mentioned
	Equipment and accessories	Reasonable, comply with Guidelines		Not mentioned
	Research materials	Reasonable, comply with Guidelines	Y	Prototype Development, Statistical tool purchase
	Rental	Reasonable, comply with Guidelines		Not mentioned
	Minor repair	Reasonable, comply with Guidelines		Not mentioned
	Special service	Reasonable, comply with Guidelines	y	Research Publications
16	Overall assessment of the proposal	In terms of quality, novelty, significance, impact, objective, scope, problem statement, literature review, references, methodology, statistics, timeliness, milestone, deliverable, cost effectiveness, budget, flowchart, project risks (technical, budget, timeline), suitability or competence of the PI, etc (strong, sound, good, poor, high, low, medium, minor weakness, major weakness, scientifically or technically flawed, all or partial assessment criteria met, etc)		
	Please summarise here:<br>The research is based on a sufficient basis, the research content is innovative, the design idea is reasonable, the research team has strong scientific research ability and high academic level.The research method is feasible, and the research results have certain social value and practical significance. However, the project has the following shortcomings:<br>1. The specific research methods and technical routes are not clearly described in the project ;			

	2.What is the relationship between the research content of this project and traditional Chinese medicine? What is the specific work of TCM researchers in the research team?

17	Recommendation	Accept proposal without revision		
		Accept proposal with minor revision	Y	
		Accept proposal with major revision		
		Reject proposal		

18	Comments for improvement and revision or Reasons for rejection:<br>Please make a minor revision for the project to make the research more reasonable and specific.

19	If necessary, recommend a revised budget:		
		Year 1, RM	Year 2, RM
	Allowance for assistant		
	Travel and subsistence		
	Equipment and accessories		
	Research materials		
	Rental		
	Minor repair		
	Special service		
	**Total amount**		

20	Remuneration for XMUMRF Peer Review
21	

XIAMEN UNIVERSITY MALAYSIA
厦門大學馬來西亞分校

Xiamen University Malaysia Research Fund (XMUMRF) Evaluation Form

**Year**	2020	**Cycle**	6

Name of evaluator	
Title/designation	
Department/Faculty/University	
Home/Office address	
Mobile phone no	
Email	
Principal Investigator (PI)	Muhammad Shahzad Aslam
Title of proposed research project	Development of Personalized Mobile Mental Health Intervention for Workplace Cyberbullying on Health practitioners

**Evaluation**				
1	Research project team		Y/N	Comment
	PI	Appropriate qualifications to conduct and complete the proposed research	Y	
	Co-researchers (if applicable)	Appropriate qualifications	Y	
2	Title of proposed research project	Appropriate for the proposed research activities, etc	Y	
		Not appropriate, too general, etc		
3	Research area/field	Appropriate or not	Y	
4	Duration	Appropriate or not	Y	
5	Abstract	Well written, informative, etc	Y	
		Poor, recommend to rewrite, etc		
6	Objective(s)	Clearly written, achievable, etc	Y	
		Ambiguous, etc		
		Too ambitious, etc		

7	Literature review/ Research background	Concise, acceptable with some relevant key references, etc	Y	
		Irrelevant, insufficient, etc		
8	Research methodology	Good, using existing methods, etc	Y	
		Good, developing new methods, etc		
		Insufficient details, etc		
9	Flowchart	Lucid, informative, etc	Y	
10	Expected results or benefits	Reasonable, achievable, etc	Y	
11	Benefits of the proposed research to XMUM, the economy, and the society	Reasonable, achievable, etc	Y	
12	Comment on the status of similar or related research in Malaysia and abroad; how is the proposed research different from them	Well written, informative, etc	N	The applicant didn't mention it at all
13	Project schedule/Gantt chart	Reasonable, achievable, etc	Y	
14	Project milestones	Lucid, achievable, etc	Y	
15	Budget			
	Allowance for assistant	Reasonable, comply with Guidelines		
	Travel and subsistence	Reasonable, comply with Guidelines		
	Equipment and accessories	Reasonable, comply with Guidelines		
	Research materials	Reasonable, comply with Guidelines	Y	
	Rental	Reasonable, comply with Guidelines		
	Minor repair	Reasonable, comply with Guidelines		
	Special service	Reasonable, comply with Guidelines	Y	
16	Overall assessment of the proposal	In terms of quality, novelty, significance, impact, objective, scope, problem statement, literature review, references, methodology, statistics, timeliness, milestone, deliverable, cost effectiveness, budget, flowchart, project risks (technical, budget, timeline), suitability or competence of the PI, etc (strong, sound, good, poor, high, low, medium, minor weakness, major weakness, scientifically or technically flawed, all or partial assessment criteria met, etc)		
	Please summarise here: With the development of network communication technology and social platform, Workshop cyberbullying on health practitioners has emerged in endlessly all over the world, and gradually developed into a serious social problem. It is very necessary to develop personalized mobile mental health intervention for workplace cyberbullying on health practitioners. The purpose of the research is clear, the technical route and research steps are feasible, the expected research results have better practicability, but the foundation of preliminary work for the research is still lacking.			

17	Recommendation	Accept proposal without revision	Y	
		Accept proposal with minor revision		Further evaluation needed: Yes / No
		Accept proposal with major revision		
		Reject proposal		

18	Comments for improvement and revision or Reasons for rejection:<br>Please comment more on the status of similar or related research of “Personalized Mobile Mental Health Intervention for Workplace Cyberbullying on Health practitioners “in Malaysia and abroad; and how is the proposed research different from them.

19	If necessary, recommend a revised budget:		
		Year 1, RM	Year 2, RM
	Allowance for assistant		
	Travel and subsistence		
	Equipment and accessories		
	Research materials		
	Rental		
	Minor repair		
	Special service		
	**Total amount**		

20	Remuneration for XMUMRF Peer Review
	Bank name (include branch for countries outside Malaysia):
	Bank address:
	Account number:
	Swift Code:
21	Signature of evaluator:
	Date:
